# Pancreatic Acinar Cells Employ miRNAs as Mediators of Intercellular Communication to Participate in the Regulation of Pancreatitis-Associated Macrophage Activation

**DOI:** 10.1155/2016/6340457

**Published:** 2016-07-28

**Authors:** Yong Zhao, Hao Wang, Ming Lu, Xin Qiao, Bei Sun, Weihui Zhang, Dongbo Xue

**Affiliations:** ^1^Department of General Surgery, The First Affiliated Hospital of Harbin Medical University, Harbin, Heilongjiang 150001, China; ^2^Department of Surgery, David Geffen School of Medicine, University of California, Los Angeles, Los Angeles, CA 90064, USA

## Abstract

Macrophage activation plays an important role in the inflammatory response in acute pancreatitis. In the present study, the activation of AR42J pancreatic acinar cells was induced by taurolithocholate treatment. The results showed that the culture medium from the activated AR42J cells significantly enhanced NF*κ*B activation in the macrophages compared to that without taurolithocholate treatment. Additionally, the precipitates obtained from ultracentrifugation of the culture media that were rich in exosomes were markedly more potent in activating macrophages compared with the supernatant fraction lacking exosomes. The results indicated that the mediators carried by the exosomes played important roles in macrophage activation. Exosomal miRNAs were extracted and examined using microarrays. A total of 115 differentially expressed miRNAs were identified, and 30 showed upregulated expression, while 85 displayed downregulated expression. Target genes of the differentially expressed miRNAs were predicted using TargetScan, MiRanda, and PicTar software programs. The putative target genes were subjected to KEGG functional analysis. The functions of the target genes were primarily enriched in MAPK pathways. Specifically, the target genes regulated macrophage activation through the TRAF6-TAB2-TAK1-NIK/IKK-NF*κ*B pathway. As the mediators of signal transduction, miRNAs and their predicted target mRNAs regulate every step in the MAPK pathway.

## 1. Introduction

Acute pancreatitis refers to a systemic inflammatory response that progresses froma local lesion (pancreas) in the peritoneal cavity. Macrophages play an important role in the inflammatory response. Macrophages produce various cytokines and inflammatory mediators, such as interleukin- (IL-) l*β*, IL-6, IL-8, and tumour necrosis factor-alpha (TNF-*α*), which cause damage tolocal tissues and distant organs. Inhibiting the activation of pancreatitis-associated macrophages not only attenuates the pathological damage to the pancreas itself but also reduces the occurrence of systemic inflammatory response syndrome and multiple organ failure to a certain extent [1].

When elucidating the mechanisms underlying the activation of pancreatitis-associated macrophages, the essential role of intercellular communication cannot be ignored [2]. Cell-cell interactions depend on signal transduction between the cells, especially intercellular communication. The precisely divided cellular functions are closely linked through intercellular communication, allowing the coordination of the overall vital activities and the synchronization of activities such as differentiation, growth, metabolism, and movement. Therefore, intercellular communication plays an important role in the physiological and pathological processes of living organisms. Chemical signal-mediated indirect cell-cell interaction constitutes the primary form of intercellular communication [3]. Cells secrete chemical signals, such as proteins, small molecules, and other organic compounds, into the extracellular environment. The chemical signals are transported to and act on recipient cells to regulate their functions.

Previous studies proposed that pancreatic enzymes released by the damaged pancreatic tissue (i.e., trypsin, elastase, carboxypeptidase A, and lipase) were the mediators of intercellular communication and thus were responsible for macrophage activation in acute pancreatitis [4, 5]. Some studies found that certain lipids extracted from ascites affected macrophage activation [6]. Other studies reported that a large number of necrotic pancreatic tissues and intestinal bacteria produced lipopolysaccharides (LPS) during acute pancreatitis episodes. LPS bind specifically to the corresponding surface receptor proteins on macrophages and promote macrophage activation. Recently, the role of microRNAs (miRNAs) as mediators of intercellular communication has become a new research hotspot. miRNAs are a group of endogenous noncoding single-stranded small RNA molecules (approximately 22 nucleotides in length) with a stem-loop structure. miRNAs are widely present in eukaryotes and inhibit the expression of their target genes at the posttranscriptional level by inducing the degradation of mRNAs or suppressing the translation of mRNAs. Recently, studies have shown that a large number of miRNAs exist outside of the cells. Extracellular miRNAs are capable of mediating intercellular communication and regulating various physiological and pathological processes [7]. One of the mechanisms that allows the existence of extracellular miRNAs involves wrapping the miRNAs in exosomes to evade degradation [8, 9]. Recently, numerous studies have shown that histiocytes and tumour cells selectively pack certain miRNAs into exosomes, which are transported to recipient cells via the circulatory system to regulate their gene expression [10].

In the present study, exosomal miRNAs obtained from activated AR42J pancreatic acinar cells were examined using microarrays. Additionally, the mechanisms that enabled the miRNAs to act as mediators of intercellular communication and activate macrophages were explored using bioinformatics methods. The objective of the present study was to provide a valuable reference for the intervention and regulation of macrophage activation during acute pancreatitis and for the prevention and treatment of acute pancreatitis.

## 2. Materials and Methods

### 2.1. Cell Culture

Rat pancreatic acinar cells (AR42J, China Centre for Type Culture Collection (CCTCC), Wuhan, China) and rat alveolar macrophages (CRL-2192 NR8383, American Type Culture Collection (ATCC), USA) were cultured in Dulbecco's modified Eagle's medium (DMEM) containing 10% foetal bovine serum (FBS), 100 U/mL of penicillin, and 100 *μ*g/mL of streptomycin at 37°C in a 5% CO_2_ incubator.

The AR42J cells were divided into two groups: the control group and the taurolithocholate (TLC) treated group. The control group was left untreated, whereas the TLC treated group was incubated with 200 *μ*M TLC for 40 min. Subsequently, the TLC-containing culture medium was discarded and replaced with fresh medium. The culture media were collected after 2 h of incubation. A portion of the collected media was used to extract exosomal RNAs, and the other portion was subjected to ultracentrifugation to isolate various components. The exosomal RNAs were examined using a miRNA microarray. The isolated medium components were added individually to macrophage culture medium at a 1 : 1 ratio to determine the effects of the individual components on NF*κ*B activation in macrophages.

### 2.2. Cell Viability Assay and Intracellular Trypsinogen Activation of AR42J Cells

Cultured cells in 96-well plate were incubated with 1 mg/mL MTT containing medium for 3 h at 37°C, and the cells were lysed with lysis buffer containing 50% of dimethyl formamide and 20% SDS. The absorbance of the dissolved MTT-derived formazan was measured at 570 nm using microtitre plate reader (Biotek, Vermont Winooski, USA). Percentage cell viability = (OD of the experiment samples/OD of the control) × 100.

10^5^ cells/mL AR42J cells were cultured for 20 min in HBS-EDTA (5 mM HEPES, 0.15 M NaCl, and 2 mM EDTA; pH 7.35) with 5 mM rhodamine 110, bis-(CBZ-L-isoleucyl-L-prolyl-L-arginine amide) dihydrochloride (BZiPAR; Molecular Probes, Invitrogen, USA). The trypsin activity was investigated by laser confocal microscopy (LSM 510 Meta, Zeiss, Germany). The experiment was repeated 3 times.

### 2.3. Examination of NF*κ*B Activity in Macrophages Using an Electrophoretic Mobility Shift Assay (EMSA)

Media harvested from AR42J cell cultures were centrifuged at 500 ×g for 5 min to remove whole cells. The macrophages were divided into 4 groups and treated as follows: Group A: complete medium collected from the control group was added to the macrophage culture at a 1 : 1 ratio; Group B: complete medium collected from the TLC treated group was added to the macrophage culture at a 1 : 1 ratio; Group C: medium harvested from the TLC treated group was centrifuged at 15,000 ×g at 4°C for 1 h, and the supernatant was added to the macrophage culture at a 1 : 1 ratio; and Group D: the precipitates that were rich in exosomes were resuspended in DMEM and added to the macrophage culture at a 1 : 1 ratio.

The macrophages were harvested after 48 h of cultivation. The NF*κ*B activity in the macrophages was examined using EMSA. Briefly, nuclear proteins were extracted using a Nuclear Extraction Kit (Thermo Fisher Scientific, 78833, USA) in accordance with the manufacturer's instructions and quantified using the bicinchoninic acid (BCA) assay (Beyotime, China). Subsequently, the samples (5 *μ*g of protein/well) were prepared according to the EMSA kit instruction manual (Thermo Fisher Scientific, 20148, USA). A 6% TBE gel was prepared, preelectrophoresed at 100 V for 1 h, and then overlaid with fresh buffer. The samples were loaded into the wells of the gel (20 *μ*L/well) and electrophoresed at 100 V until the bromophenol blue dye migrated approximately 2/3 of the length of the gel. Then, the samples were electroblotted onto a nylon membrane (0.45 *μ*m pore size, Millipore, USA) with a 380 mA constant current for 30 min and cross-linked for 15 min at 254 nm using an ultraviolet cross-linker. The membrane was blocked for 15 min. After blocking, the membrane was completely immersed in streptavidin-horseradish peroxidase (HRP) solution (1 : 300 dilution, Thermo Fisher Scientific, 21130, USA) and incubated at room temperature for 15 min with gentle shaking. The membrane was washed 5 times (5 min per wash) and equilibrated for 5 min in Substrate Equilibration Buffer. The enhanced chemiluminescence (ECL) reagent was added to the membrane and allowed to react for 2 min. Then, the membrane was exposed to an autoradiographic film. The film was developed and fixed. The experiment was repeated 3 times.

### 2.4. Extraction of Exosomal RNAs from the Culture Media of Rat Pancreatic Acinar Cells

Exosomal RNAs were extracted using a Norgen Exosome RNA Isolation Kit (Norgen Biotek, 42800, Canada) in accordance with the manufacturer's instructions. In 50 mL tubes, 0.2 mL of PS Solution A and 1.8 mL of PS Solution B were added to every 1 mL of the medium sample after the addition of *β*-mercaptoethanol. The mixture was incubated at 60°C for 10 min. Subsequently, 3 mL of 100% ethanol was added to each tube. After centrifugation at 1,000 rpm for 30 s, the resulting supernatants were carefully decanted. The slurry pellets were mixed thoroughly with 0.3 mL of PS Solution C and incubated at 60°C for 10 min. After incubation, 0.3 mL of 100% ethanol was added to the mixture. 650 *μ*L of the mixture was loaded onto the Mini Filter Spin column and centrifuged at 14,000 rpm for 1 min. The flow-through in the collection tube was discarded, and the spin column was reassembled with its collection tube. After the addition of 400 *μ*L of Wash Solution, the column was centrifuged at 14,000 rpm for 1 min. The washing step was repeated 2 more times. The empty column was centrifuged for an additional 3 min at 14,000 rpm to completely remove any residual Wash Solution. The collection tube was discarded and the spin column was transferred to a fresh 1.7 mL Elution tube. After the addition of 100 *μ*L of Elution solution, the column was centrifuged at 2,000 rpm for 2 min, followed by centrifugation at 14,000 rpm for 3 min.

### 2.5. *μ*Paraflo™ MicroRNA Microarray Assay

Microarray assay was performed using a service provider (LC Sciences, USA). The assay started from 6 *μ*g total RNA sample was 3′-extended with a poly(A) tail using poly(A) polymerase. An oligonucleotide tag was then ligated to the poly(A) tail for later fluorescent dye staining. Hybridization was performed overnight on a *μ*Paraflo microfluidic chip using a microcirculation pump (Atactic Technologies, USA) [11, 12]. On the microfluidic chip, each detection probe consisted of a chemically modified nucleotide coding segment complementary to target microRNA (from miRBase, http://www.mirbase.org/) or other RNAs (control or customer defined sequences) and a spacer segment of polyethylene glycol to extend the coding segment away from the substrate. The detection probes were made by* in situ* synthesis using PGR (photogenerated reagent) chemistry. The hybridization melting temperatures were balanced by chemical modifications of the detection probes. Hybridization used 100 L 6xSSPE buffer (0.90 M NaCl, 60 mM Na_2_HPO_4_, and 6 mM EDTA, pH 6.8) containing 25% formamide at 34°C. After RNA hybridization, tag-conjugating Cy3 dye was circulated through the microfluidic chip for dye staining. Fluorescence images were collected using a laser scanner (GenePix 4000B, Molecular Device, USA) and digitized using Array-Pro image analysis software (Media Cybernetics). Data were analyzed by first subtracting the background and then normalizing the signals using a LOWESS filter (Locally-Weighted Regression) [13].

### 2.6. Prediction of the Differentially Expressed miRNA Target Genes

The target genes of the significantly differentially expressed miRNAs were predicted using TargetScan (http://www.targetscan.org/), MiRanda (http://www.microrna.org/microrna/home.do), and PicTar (http://pictar.mdc-berlin.de/) software programs.

The putative target genes predicted by the 3 software programs were screened separately using the scoring criteria of the corresponding software. In the TargetScan algorithm, putative target genes with a context score percentile less than 50 were excluded. In the MiRanda algorithm, putative target genes with a Max-Energy value greater than −10 were discarded. In PicTar, putative target genes with a ddG score higher than −5 were removed. The final target genes of the differentially expressed miRNAs were established by identifying the intersection of the target genes predicted by the 3 software programs.

### 2.7. Annotation and Enrichment Analysis of the Functions and Signalling Pathways Mediated by the Differentially Expressed miRNA Target Genes

KEGG (Kyoto Encyclopaedia of Genes and Genomes) is the primary public pathway-related database. Pathway enrichment analysis employs the KEGG pathway as the basic unit. Using a hypergeometric test against a whole genome background, pathway enrichment analysis identifies significantly enriched pathways associated with differentially expressed genes. *P* values were calculated based on the following formula:

(1) $$\begin{array}{c} P=1-\overset{m-1}{\underset{i=0}{\sum }}\frac{\left(\begin{array}{c} M\\ i \end{array}\right)\left(\begin{array}{c} N-M\\ n-i \end{array}\right)}{\left(\begin{array}{c} N\\ n \end{array}\right)}, \end{array}$$

where *N* represents the number of genes with pathway annotation, *n* is the number of differentially expressed genes in *N*,  *M* represents the number of all genes annotated to a specific pathway, and *m* is the number of differentially expressed genes in *M*. The threshold *P* value was set to 0.05. A KEGG pathway was considered significantly enriched in the differentially expressed genes if the calculated *P* value was equal to or less than 0.05.

A diagram depicting the miRNA target mRNA regulatory network was constructed using Cytoscape (V2.8.3). Thumbnail images of the relevant KEGG pathways were obtained from the KEGG website (http://www.kegg.jp/kegg/pathway.html). The differentially expressed miRNAs and their target mRNAs were indicated.

### 2.8. Real-Time Reverse Transcription Polymerase Chain Reaction (RT-PCR) for Verification of Expression Levels of Some miRNAs and Their Target mRNAs

Total RNA was extracted from each group of cells according to the instruction manual of the RNA extraction kit (Qiagen, Hilden, Germany) and reverse transcribed into complementary DNA (cDNA) according to the manufacturer's instructions of HiFi-MMLVcDNA reverse transcription kit (CWbio. Co. Ltd., Cat#CW0744, Beijing, China). The cDNAs were then subjected to fluorescence-based quantitative PCR using primers (Bio-Serve Co. Ltd., Shanghai, China) specific for the target mRNAs and miRNAs. The primer sequences are summarized in Table S1 in Supplementary Material available online at http://dx.doi.org/10.1155/2016/6340457. Fluorescence-based quantification was achieved using the ABI Prism 7500 Real-Time PCR system (Applied Biosystems, Foster City, CA, USA). All data were subjected to relative quantitative analysis using the 2^−ΔΔCt^ method. Expression levels of mRNA from TLC treated AR42J cells were expressed as a multiple or fraction of the control group, which is considered arbitrarily as 1. The experiment was repeated 3 times.

## 3. Results

### 3.1. The Examination of NF*κ*B Activation in Macrophages after the Addition of Various Conditioned Media

**Figure 1:**
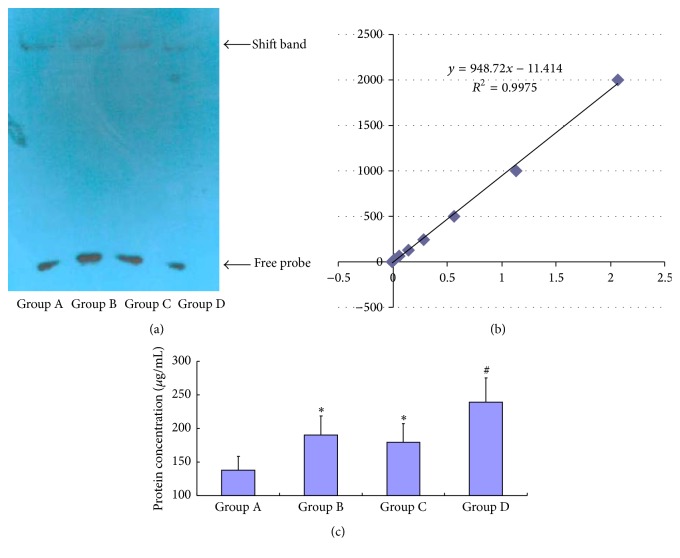
The examination of NF*κ*B activation in macrophages. (a) Scanned EMSA gel images. (b) Standard curves. (c) Statistical graph of the EMSA results. *∗* indicates statistically significant differences compared with Group A (*P* ≤ 0.05). # indicates statistically significant differences compared with groups B and C (*P* ≤ 0.05).

The retarded bands representing the cross-linked DNA-protein complexes were detected on the scanned images of the EMSA gel (Figure 1(a)). The EMSA results were analyzed (Figure 1(c)). The NF*κ*B activity was significantly increased in Group B (190.18 ± 28.42 *μ*g/mL) and Group C (179.75 ± 27.63 *μ*g/mL) compared to the control group (137.93 ± 20.78 *μ*g/mL) (*P* ≤ 0.05). Additionally, the macrophages in Group D exhibited a significantly higher level of NF*κ*B activation (239.27 ± 35.94 *μ*g/mL) compared with Group B and Group C (*P* ≤ 0.05).

### 3.2. Microarray Analysis of the Exosomal miRNAs Isolated from the Rat Pancreatic Acinar Cell Culture Media

In the present study, the TLC treated AR42J cells appeared as brighter green fluorescence than the control cells which proved that significant elevated intracellular trypsinogen activation. The quality of the extracted exosomal RNAs was assessed by real-time polymerase chain reaction (PCR) analysis of the indicators has-miR-16 and has-miR-192. Based on the range of the threshold values (Ct) of the indicators reported in the literature, we determined that the extracted exosomal RNAs met the quality standards. Following hybridization, the microarray was scanned using a scanner. The data were extracted, LOWESS filtered and normalized, and then subjected to differential expression analysis. The quality control (QC) parameters were as follows: in the TLC treated group, the coefficient of variation (CV) = 6.28 and the QC standard was ≤15%, and in the control group, the CV = 7.68 and the QC standard was ≤15%. CV was defined as the ratio of the standard deviation (SD) to the mean and was expressed as a percentage. The CV was calculated using the follow formula: CV = SD/Mean × 100%. In the miRNA microarray analysis (LC Sciences), the stability of the microarray and the technique were evaluated by calculating the CV of the spot intensities of the repeat probes. The QC criterion recommended by LC Sciences was a CV value less than 15%.

**Figure 2:**
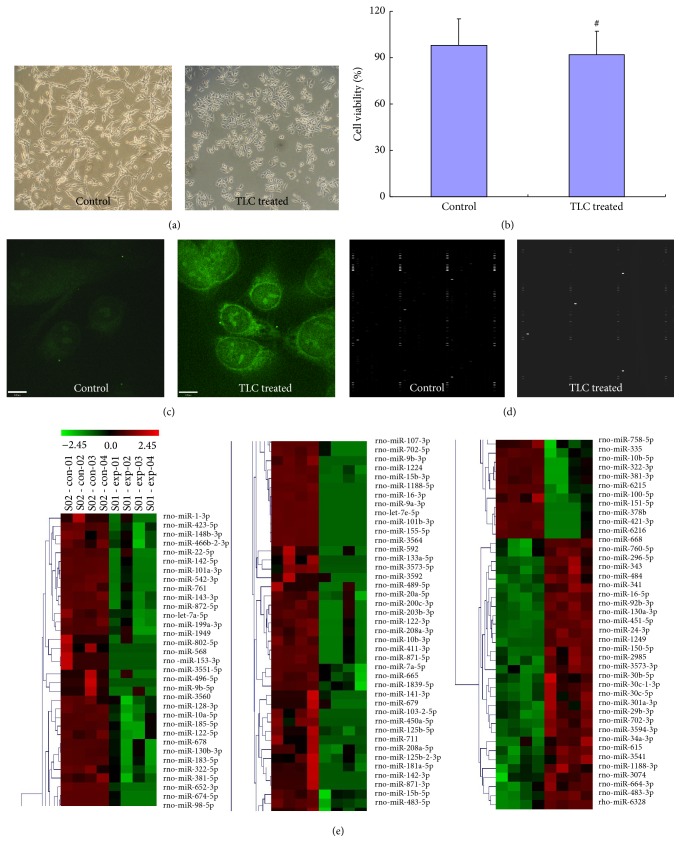
The microarray analysis of the exosomal miRNAs isolated from the culture medium of rat pancreatic acinar cells. (a) Microscopic images of cultured rat pancreatic acinar cells-AR42J cells. (b) MTT assay for detection of cell viability of AR42J cells; there is no significant difference between the two groups, ^#^ *P* > 0.05. (c) Laser confocal microscopic images of AR42J cells labelled by BZiPAR for assessing intracellular trypsinogen activation. (d) Microarray hybridization profiles of exosomal miRNAs isolated from the culture medium of AR42J cells. (e) Cluster analysis diagram. Green denotes miRNAs expressed at low levels in the corresponding samples, whereas red denotes the miRNAs expressed at high levels in the corresponding samples.

In the present study, 115 differentially expressed miRNAs were identified using the miRNA microarray. Among the differentially expressed miRNAs, 30 were upregulated and 85 were downregulated. The results of the cluster analysis are shown in Figure 2(c). The miRNA clusters are shown in the left panel of Figure 2(c). miRNAs with similar expression profiles were clustered together (bracketed together). The scale bar placed above the miRNA clusters represented the range of the *Z* value. The formula used to calculate the *Z* value was provided below the clusters. The *Z* value was calculated using the following formula: *Z*_sample-*i*_ = [(log 2(Signal_sample-*i*_)) − Mean(log 2(Signal)  of  all  samples)]/[Standard  deviation(log 2(Signal)  of  all  samples)].

### 3.3. KEGG Pathway Enrichment Analysis of the Target Genes of the Differentially Expressed miRNAs

**Figure 3:**
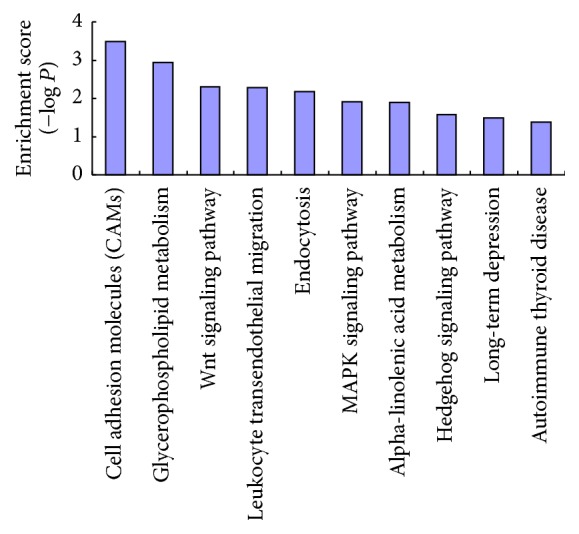
KEGG pathway analysis of genes that were regulated by differentially expressed miRNA. The enrichment score of each pathway was computed (enrichment score = −log *P* value).

As shown in Figure 3, 10 KEGG pathways enriched with the functions of the differentially expressed miRNAs were identified using a *P* value threshold of 0.05. The pathways included cell adhesion molecules (CAMs), glycerophospholipid metabolism, the Wnt signalling pathway, leukocyte transendothelial migration, endocytosis, the MAPK signalling pathway, alpha-linolenic acid metabolism, the Hedgehog signalling pathway, long-term depression, and autoimmune thyroid disease. After conducting a comprehensive literature search, we found that the MAPK signalling pathway (ranked 6 among the KEGG pathways) was closely related to the activation of macrophages and NF*κ*B. Therefore, the MAPK signalling pathway was the focus of our subsequent analyses.

### 3.4. The Regulatory Effect of the miRNAs and Their Target mRNAs in the MAPK Pathway on NF*κ*B Activation

**Figure 4:**
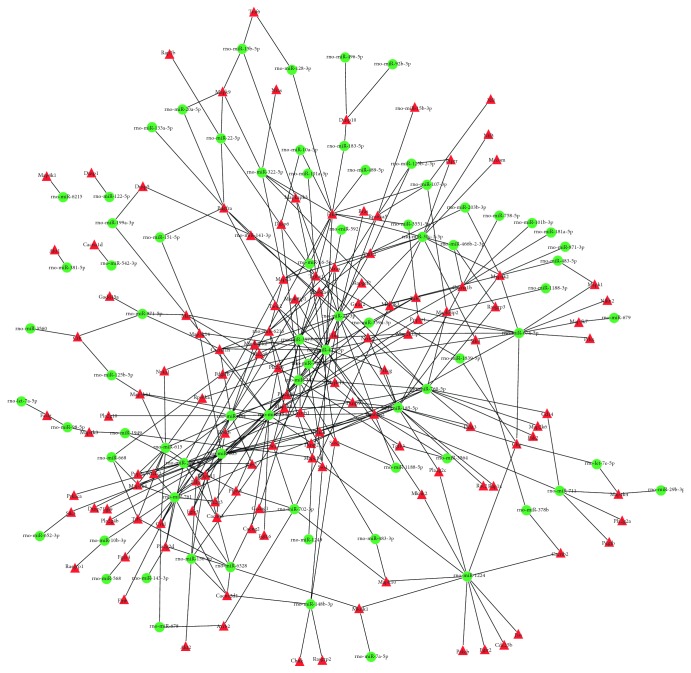
Interaction network of differentially expressed miRNAs and their regulated target genes. Green ellipse shape nodes refer to differentially expressed miRNA, and red triangle shape nodes refer to their regulated target genes.

Using Cytoscape software, we constructed a network diagram depicting the mutual regulation between the miRNAs and mRNAs in the KEGG pathways (Figure 4). We found that 71 differentially expressed miRNAs were responsible for the regulation of 129 mRNAs. The miRNAs and mRNAs constituted 338 interacting pairs.

**Figure 5:**
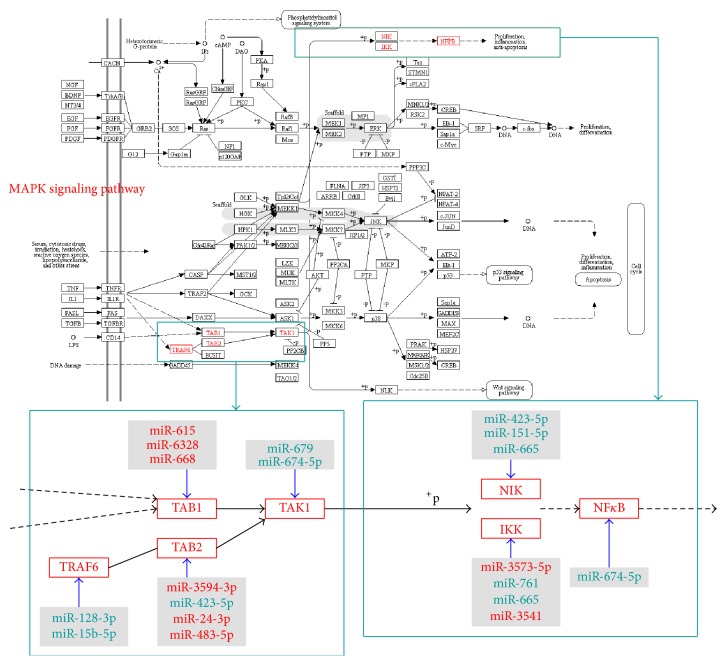
Thumbnail image of MAPK signalling pathway. The genes labelled red were genes that were regulated by differentially expressed miRNA. The miRNAs labelled red were the upregulated miRNAs, and the miRNAs labelled green were the downregulated miRNAs.

Thumbnail images of the MAPK pathways provided by the KEGG website were employed to display the KEGG pathways and the distribution of the significantly differentially expressed genes in the KEGG pathways and to discover and provide useful information (Figure 5). The predicted target genes of the differentially expressed miRNAs were denoted with red boxes. As shown in Figure 5, the target genes of the differentially expressed miRNAs regulated NF*κ*B activation through the TNF receptor-associated factor 6 (TRAF6) (TGF-beta activated kinase 1/MAP3K7 binding protein 1 (TAB1) or TGF-beta activated kinase 1/MAP3K7 binding protein 2 (TAB2)) transforming growth factor beta activated kinase 1 (TAK1) (NF*κ*B inducing kinase (NIK) or I*κ*B kinase (IKK)) NF*κ*B pathway. Additionally, specific differentially expressed miRNAs participated in the regulation of these target genes, thereby affecting NF*κ*B activation. The regulatory pairs were as follows: miR-128-3p and miR-15b-5p regulate TRAF6; miR-615, miR-6328, and miR-668 regulate TAB1; miR-3594-3p, miR-423-5p, miR-24-3p, and miR-483-5p regulate TAB2; miR-679 and miR-674-5p regulate TAK1; miR-423-5p, miR-665, and miR-151-5p regulate NIK; miR-3573-5p, miR-761, miR-665, and miR-3541 regulate IKK; and miR-674-5p regulates NF*κ*B.

### 3.5. Expression of miRNAs and Their Target mRNA Verified by Real-Time RT-PCR

**Figure 6:**
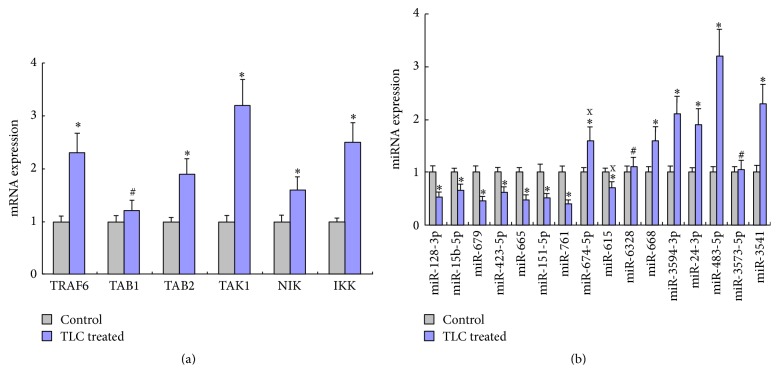
Expression levels of mRNA or miRNA detected by real-time RT-PCR. ^*∗*^Compared to control group, *P* < 0.05. ^#^Compared to control group, *P* > 0.05. ^x^The expression level was incompatible between the chip and real-time RT-PCR detection.

The results of real-time RT-PCR verified that the expression level of miR-668, miR-3594-3p, miR-24-3p, miR-483-5p, miR-3541, and TRAF6, TAB2, TAK1, NIK, and IKK was upregulated in TLC treated group (*P* < 0.05), while the expression level of miR-128-3p, miR-15b-5p, miR-679, miR-423-5p, miR-665, miR-151-5p, miR-761, was miR-615 downregulated (*P* < 0.05); see Figure 6. The real-time RT-PCR results showed that the expression of miR-674-5p, miR-615, miR-6328, and miR-3573-5p was not in accordance with the results of chip detection. And TAB1 showed no significant elevated (*P* > 0.05).

## 4. Discussion

Currently, the mechanisms underlying the acute pancreatitis-induced systemic inflammatory response syndrome are commonly thought to include the overactivation of leukocytes and the release of excessive amounts of cytokines. Monocytes/macrophages are thought to play a crucial role in the above process [14]. Acute pancreatitis begins with the activation of trypsinogen. The damaged pancreatic cells release a variety of proinflammatory mediators that first stimulate pancreatic macrophages, followed by peritoneal macrophages and then macrophages in the liver, lungs, and gastrointestinal tract. The overactivated monocytes/macrophages secrete more proinflammatory cytokines, which enter the blood circulation and contribute to the development of the systemic inflammatory response syndrome. Therefore, an exploration of the activation mechanisms of pancreatitis-associated macrophages may achieve a valuable breakthrough in the clarification of the pathogenesis of acute pancreatitis and the systemic inflammatory response related to acute pancreatitis [15]. Inhibiting macrophage activation has been shown to attenuate the pathological damage to the pancreas and improve prognoses to a certain extent. Shifrin et al. [16] established a mouse model of viral infection-induced acute pancreatitis and used dichloromethylene diphosphonate to deplete macrophages. The results showed that macrophage depletion increased the mouse survival rate. The study conducted by Souza et al. [17] found that the TNF-*α*, IL-6, and IL-10 serum levels were reduced in mice with acute pancreatitis after clearance of macrophages by peritoneal lavage. Gloor et al. [18, 19] reported that selective clearance of macrophages in different organs led to improvements in both organ and systemic lesions. Thus, cell-level therapies that target macrophages have become a hotspot and the regulation of macrophage functions has become a new research direction in the treatment of acute pancreatitis.

NF*κ*B plays an important role in the activation of acute pancreatitis-related macrophages [20]. The NF*κ*B and p38 MAPK signalling pathways were shown to be activated in circulating monocytes in a taurine-induced acute pancreatitis model [21]. Another study showed that NF*κ*B induced the activation of virulizin, which further promoted the production of TNF-*α* by peritoneal macrophages [22]. The study conducted by Ma et al. [23] found that the administration of resveratrol decreased the expression of NF*κ*B and inducible nitric oxide synthase (iNOS) in* in vitro* cultured peritoneal macrophages and reduced the TNF-*α*, IL-1, and nitric oxide (NO) levels in the culture medium and serum. Gutierrez et al. [6] found that coculture of macrophages with the ascites from model mice with acute pancreatitis resulted in macrophage activation. However, the addition of the NF*κ*B inhibitor pyrrolidine dithiocarbamate (PDTC) to the culture medium terminated macrophage activation. In the present study, the activation of pancreatic acinar cells was induced by TLC treatment. When the activated pancreatic acinar cell-conditioned culture medium was added to the macrophage culture, the results showed that the culture medium from the activated pancreatic acinar cells significantly enhanced the level of NF*κ*B activation in the macrophages compared to culture medium from cells without TLC treatment. Thus, the activated pancreatic acinar cells promoted the activation of macrophages through the activity of certain mediators. To identify these mediators, culture media were subjected to ultracentrifugation, and we observed the effects of the supernatant fractions lacking exosomes and of the precipitates, which were rich in exosomes. The results showed that the precipitates were markedly more potent in activating macrophages than the supernatant fractions, indicating that the exosomes and the mediators carried by the exosomes played an important role in macrophage activation. Extracellular RNAs carried by exosomes and other vesicles are capable of regulating intracellular RNA signals in recipient cells. Therefore, extracellular RNA has become a novel therapeutic target.

To explore these mediators of signal transduction, the extracted exosomal RNAs were examined using a miRNA microarray. The results showed significant differences in the miRNA expression profiles between the exosomes secreted by the TLC treated pancreatic acinar cells and the exosomes secreted by the untreated pancreatic acinar cells. A total of 115 differentially expressed miRNAs were identified, among which 30 showed upregulated expression and 85 displayed downregulated expression. The differentially expressed miRNAs carried by the exosomes were integrated into the recipient cells with the exosomes and affected recipient cell functions. At present, studies have demonstrated that miRNAs exert a regulatory effect on macrophage activation. Taganov et al. [24] stimulated macrophages with LPS and found that the expression of certain miRNAs was increased. Zhang et al. [25] discovered that 109 miRNAs were differentially expressed between murine bone marrow-derived M1 and M2 macrophages, suggesting that miRNAs were involved in the regulation of macrophage polarization. To elaborate the functions of the miRNAs that acted as mediators of signal transduction, we predicted the target genes of the miRNAs and annotated the functions of the target genes in the present study. Additionally, we performed KEGG pathway enrichment analysis. Among the significantly enriched pathways, the MAPK signalling pathways were the most closely related to the activation of macrophages and NF*κ*B.

There is a large amount of evidence that MAPKs are involved in the activation of a number of proinflammatory nuclear transcription factors, including NF*κ*B, and that the MAPK signalling pathways interact with the NF*κ*B signalling pathway [26–29]. We mapped the miRNAs and miRNA-regulated target mRNAs to the MAPK signalling pathways and found that the differentially expressed miRNAs primarily acted on the TRAF6-TAB2-TAK1-NIK/IKK-NF*κ*B pathway. In the above pathways that regulate NF*κ*B activation, multiple differentially expressed miRNAs target and regulate every step of the pathway, thereby ultimately affecting NF*κ*B and macrophage activation.

In the MAPK pathways, binding of LPS to toll-like receptor 4 (TLR4) enables transmission of activating signals downstream via the toll/IL-1 receptor (TIR) domain, resulting in the sequential activation of interleukin-1 receptor-associated kinase (IRAK), TRAF6, TAK1, IKK, and other key molecules. These key molecules activate NF*κ*B, thereby enhancing the transcription and expression of the proinflammatory cytokines IL-1, IL-6, and TNF-*α* and activating monocytes/macrophages [13]. The results of the present study revealed that the differentially expressed miRNAs carried by exosomes were capable of modulating various steps in the MAPK pathways.

TRAF6 is a member of the tumour necrosis factor receptor- (TNFR-) associated factor family and a key mediator that is capable of simultaneously transducing signals from the TNFR superfamily and the IL-1R/TLR superfamily [30–32]. As an important adapter molecule, TRAF6 regulates the expression of a series of proinflammatory factors through NF*κ*B, including TNF-*α*, IL-2, IL-6, IL-8, and IL-12 [33, 34]. The results of the present study revealed that TRAF6 was a target of exosome-carried miR-128-3p and miR-15b-5p.

TAB1/2 are adaptor proteins for TRAF6. TRAF6 effectively activates its downstream protein TAK1 with the assistance of TAB1/2. The present study found that, among the differentially expressed miRNAs, miR-423-5p regulated TAB2.

TAK1 is a member of the MAP kinase kinase kinase (MKKK) family. TAK1, which is a special activating factor for TAB1, and TRAF6 jointly activate the downstream kinases NIK and IKK [35]. TAK1 has been demonstrated to play an important role in the TLR4-dependent LPS signalling system, which activates NF*κ*B [36]. Additionally, TAK1 exerts a regulatory effect on MKKK during TLR/IL-1 receptor-initiated p38MAPK activation [37, 38]. The present study found that TAK1 was the target of downregulated miR-679.

Membrane receptors transmit signals to NIK, which then selectively stimulates the IKK complex composed of IKK*α* and IKK*β* and catalyses the phosphorylation of inhibitor of kappa B (I*κ*B). As a result, the key protein/immediate-early nuclear transcription factor capable of promoting cell growth (NF*κ*B) is released in an active functional state to promote cellular DNA transcription and enhance the expression of related genes [39, 40]. The present study found that downregulated miR-423-5p, miR-665, and miR-151-5p targeted NIK, whereas downregulated miR-761 and miR-665 exerted their regulatory effect by targeting IKK.

## 5. Conclusions

In summary, activated pancreatic acinar cells regulate macrophage activation and NF*κ*B activation by secreting exosomes carrying differentially expressed miRNAs. During the process of NF*κ*B activation in macrophages, the differentially expressed miRNAs act as mediators to regulate various steps of the TRAF6-TAB2-TAK1- NIK/IKK-NF*κ*B pathway (one of the MAPK signalling pathways). Therefore, the present study provides novel ideas for future studies of the regulation of pancreatitis-associated macrophage activation and the alleviation of the progression of acute pancreatitis.

## Supplementary Material

RT-PCR was carried out for verification of expression levels of 16 differentially expressed miRNAs screened using microRNA microarray assay and 6 target mRNAs predicted using bioinformatics analysis. The primer sequences used for detecting these miRNAs and mRNAs were summarized in the table list below
