# Supplementary material for: Pancreatic Acinar Cells Employ miRNAs as Mediators of Intercellular Communication to Participate in the Regulation of Pancreatitis-Associated Macrophage Activation
Source: Mediators Inflamm. 2016 Jul 28;2016:6340457. doi: 10.1155/2016/6340457 (PMC4980583; doi:10.1155/2016/6340457)
Supplement: Supplementary file 1 — RT-PCR was carried out for verification of expression levels of 16 differentially expressed miRNAs screened using microRNA microarray assay and 6 target mRNAs predicted using bioinformatics analysis. The primer sequences used for detecting these miRNAs and mRNAs were summarized in the table list below [file 6340457.f1.pdf]

Table S1 Oligonucleotide primer sets for real-time PCR

name		Sequence(5'¬3')
TRAF6	F	TCCCAGTTGGTTGTGAAGT
	R	GACCCAAGTTTCCGTGCC
TAB1	F	GACCCAAGTTTCCGTGCC
	R	ATAGCCATTGAAGACCC
TAB2	F	GAGACGGCTGCCCAAGT
	R	TCGCCTGCTCAGGTCTATC
TAK1	F	AAAGGCTTCCGCAGTTAG
	R	CGGTGGTGCTTGTTGATG
NIK	F	ACACTCGCTTGTTTGAGGA
	R	GGCTGTGCCTTTGGTGAT
IKK	F	TTCCAGCTGAGGAAAGTGTG
	R	TTCCAGCTGAGGAAAGTGTG
β-actin	F	GGAGATTACTGCCCTGGCTCCTAGC
	R	GGCCGGACTCATCGTACTCCTGCTT
miR-128-3p		UCACAGUGAACCGGUCUCUUU
miR-15b-5p		UAGCAGCACAUCAUGGUUUACA
miR-679		CAAGGUCUUCCUCACAGUAGC
miR-423-5p		UGAGGGGCAGAGAGCGAGACUUUU
miR-665		ACCAGGAGGCUGAGGUCCCUUA
miR-151-5p		UCGAGGAGCUCACAGUCUAGU
miR-761		GCAGCAGGGUGAAACUGACACA
miR-674-5p		GCACUGAGAUGGGAGUGGUGUA
miR-615		GGGGGUCCCCGGUGCUCGGAUC
miR-6328		AGGCCUGCUCUGAGCCCCCGC
miR-668		UGUCACUCGGCUCGGCCCACUACC
miR-3594-3p		CCCAGGGCAGAGCAGUGUGAA
miR-24-3p		UGGCUCAGUUCAGCAGGAACAG
miR-483-5p		AAGACGGGAGAAGAGAAGGGAG
miR-3573-5p		UGAGGGGCAGUGAUAGAAAGGA
miR-3541		UCCCUCCCCCUCACUGCA
U6		AAGGATGACACGCAAATTCG
